# Health, Economic, and Social Impacts of Substandard and Falsified Medicines in Low- and Middle-Income Countries: A Systematic Review of Methodological Approaches

**DOI:** 10.4269/ajtmh.22-0525

**Published:** 2023-06-20

**Authors:** Raimat Korede Salami, Sara Valente de Almeida, Adrian Gheorghe, Sarah Njenga, Wnurinham Silva, Katharina Hauck

**Affiliations:** ^1^ Department of Infectious Disease and Epidemiology, School of Public Health, Imperial College London, London, United Kingdom;; ^2^ Center for Life Course Health Research, Faculty of Medicine, University of Oulu, Oulu, Finland

## Abstract

Little is known about the adverse health, economic, and social impacts of substandard and falsified medicines (SFMs). This systematic review aimed to identify the methods used in studies to measure the impact of SFMs in low- and middle-income countries (LMICs), summarize their findings, and identify gaps in the reviewed literature. A search of eight databases for published papers, and a manual search of references in the relevant literature were conducted using synonyms of SFMs and LMICs. Studies in the English language that estimated the health, social, or economic impacts of SFMs in LMICs published before June 17, 2022 were considered eligible. Search results generated 1,078 articles, and 11 studies were included after screening and quality assessment. All included studies focused on countries in sub-Saharan Africa. Six studies used the Substandard and Falsified Antimalarials Research Impact model to estimate the impact of SFMs. This model is an important contribution. However, it is technically challenging and data demanding, which poses challenges to its adoption by national academics and policymakers alike. The included studies estimate that substandard and falsified antimalarial medicines can account from 10% to ∼40% of total annual malaria costs, and SFMs affect rural and poor populations disproportionately. Evidence on the impact of SFMs is limited in general and nonexistent regarding social outcomes. Further research needs to focus on practical methods that can serve local authorities without major investments in terms of technical capacity and data collection.

## INTRODUCTION

The United Nations established universal health coverage (UHC) in all member countries as one of the Sustainable Development Goals for 2030, with a specific focus on universal access to safe and essential medicines.^1^ The presence of substandard and falsified medicines (SFMs) around the world threatens the achievement of this goal, and according to the WHO, ∼10% of all medical products in low- and middle-income countries (LMICs) are either substandard or falsified.^2^^,^^3^

Historically, SFMs have been described with different terminologies such as counterfeit, fake, and poor quality, all of which are still used somewhat interchangeably in the literature covered in this review. In 1999, the WHO produced a report^4^ that defined counterfeit drugs specifically as “a medicine which is deliberately and fraudulently mislabeled with respect to identity and source.” In the same report, the WHO advised governments to establish national drug regulatory authorities to be responsible for the licensing of legitimate drugs and identification of SFMs. Substandard medicines are now defined by the WHO as “authorized medical products that fail to meet either their quality standards or specification, or both”; falsified medicines are defined as “medical products that deliberately/fraudulently misrepresent their identity, composition or source.”^3^ Research on this subject increased significantly since then and demonstrated that the existence of SFMs needs to be tackled as a serious public health challenge.^3^^,^^5^^–^^10^

The channels through which SFMs can have an impact on a population’s health, economic, and social status are manifold.^11^ Patients who do not receive effective treatment can remain ill for longer periods and experience increased morbidity and mortality, with detrimental effects to both individuals and communities.^3^^–^^9^^,^^11^^,^^12^ In the case of infectious diseases, treatment failure may also increase the risk of transmission to other individuals.^12^ At the social level, there is a wide range of possible impacts of the prevalence of SFMs on values, relationships, and dynamics that have not yet been identified or quantified. Patients and informal caregivers may have to spend additional time and financial resources on health and social care. As a result, patients and carers are less productive and their personal income is reduced, with potential adverse effects on the economic welfare of communities and countries. This means an economic loss for all agents involved—patients, their families, the health system, the pharmaceutical industry, and national productivity. The prevalence of SFMs can also lead to loss of confidence in health systems and medical staff, with adverse impacts on care-seeking behavior. Moreover, scarce medical resources are wasted and are less available for the financing of good-quality medicines.^10^^,^^12^ This situation frustrates both patients and providers. Patients may try to procure alternative treatments that are not as effective; health workers may feel that the value of their work is unrecognized. Substandard and falsified medicines are often consumed disproportionally by poorer households; therefore, they can aggravate existing inequalities in health and economic status.^13^ It is important to understand the magnitude and direction of these impacts to inform public policies and design them to protect, in particular, the most vulnerable patients from SFMs.

In the context of assessing the adverse impacts of SFMs, during past years several studies were conducted to address this issue, most of which were in sub-Saharan Africa (SSA) and focused on substandard and falsified (SF) antimalarial medicines.^6^^,^^14^ Mathematical modeling has been shown to be a very useful tool in these studies. However, because of the scarcity of reliable population-based data from LMICs, authors often need to simulate a variety of scenarios over a range of SFM prevalence to achieve a broad picture of the potential impacts for society. According to estimates, our systematic review found that SF antimalarial medicines can account for ∼10% to 40% of total annual malaria costs, and SFMs affect rural and low-socioeconomic status (SES) populations disproportionately. To the best of our knowledge, there is no agreed or standard method that allows countries and institutions to estimate (even broadly) the magnitude of SFMs in national pharmaceutical markets. Reliable empirical estimates of the impacts of SFMs are crucial to provide rigorous evidence for policies that improve surveillance, regulation, and governance of the pharmaceutical supply chain.^5^

Our systematic review aims to identify the existing methodological approaches used to estimate the health, economic, and social impact of SFMs in LMICs. It also presents a summary of the results found with each method and current gaps in the literature, while outlining the geographic settings and medicine classes included in existing research of SFM impacts.

## MATERIALS AND METHODS

This systematic review was conducted in accordance with the preferred reporting items for systematic reviews and meta-analysis guidelines.^15^

### Search strategy.

A preliminary search was conducted in early March 2021 to find medical subject headings entry terms associated with “substandard and falsified medicines” and “low-middle-income countries.”^16^ After this, the full review started in late March 2021 and was updated in June 2022. Details on the search strategy are presented in Supplemental Tables 1–4. The Ovid search engine was used to search the Medline, Embase, Econlit, and Global Health databases. Scopus, Web of Science, Open Grey Literature Database (http://www.opengrey.eu/), and WHO Essential Medicines and Health Product Information Portal were also searched.^17^^,^^18^ Because we use open-source registered guidelines for systematic literature reviews, the specific protocol for this systematic review was not registered.

### Selection and synthesis.

Search results were downloaded from databases and stored in EndNote software (v.20.2, Clarivate, PA) before being uploaded to Covidence software systematic review software (Veritas Health Innovation, Australia) for screening. Titles and abstracts were screened against inclusion and exclusion criteria. When this was inconclusive, the full text was evaluated. All work included had to meet all inclusion criteria and none of the exclusion criteria. Eligibility criteria consisted of primary research papers in English estimating the health and/or economic and/or social impact of SF drugs in LMICs. Articles published in languages other than English or that were not primary original research (reviews, commentaries, and letters) were excluded. Any studies with settings not defined as LMICs by the World Bank at the time of the search (both in March 2021 and June 2022) were excluded.^18^ We included work that estimated impacts of prevalence after laboratory testing, and that focused on health and socioeconomic outcomes and impacts of SFMs. Studies that solely estimated the prevalence of SFMs or tested protocols for SFMs (real laboratory studies/laboratory procedures) were excluded, as well as studies using animal populations, those measuring the effects of herbal medicines, or those focusing on legislative issues surrounding medicine quality. Our inclusion criteria allowed for studies using SFM synonyms such as counterfeit, poor quality, or fake. Studies in which effects related to SFMs or non-SFMs were not clear were excluded. Literature on physiological impacts of SFMs (e.g., adverse drug reactions) were not included.

This systematic review is limited by the existing evidence on the impacts of SF medical products,^19^ which focus our search on medicines, excluding other medical products such as vaccines or diagnostic kits, as pointed out by the 2020 WHO report.^20^^–^^22^ Vaccines are a specific medical product, distinct from drugs, typically provided at the national level and with heavily regulated distribution and commercialization.^23^ This means that all the steps involved in the research process, from defining which products to sample, how to sample them, where to sample them, how to test them, and the laboratory requirements to test vaccines are distinct from those of drugs. References of included articles and relevant systematic reviews were hand-searched and eligible citations were included.^6^^–^^8^

The following data were extracted for analysis from the included articles:

1. Basic characteristics of the studies, including year of data collection, year of publication, and geographic setting
2. Drug class or molecules under study and the respective diseases they treat
3. Analysis strategy (e.g., modeling or statistical analysis) and data sources
4. Health outcomes, including individual and aggregated health measures that indicate the impact on morbidity and/or mortality (e.g., number of infected individuals, hospitalizations, deaths, disability-adjusted life years, years of life lost)
5. Economic and social impacts, including direct and indirect costs associated with monitoring, controlling, and treating a given disease on the health system, patients, or society (including medication and hospitalization costs, travel and waiting costs to patients, diagnostic costs, and productivity losses). Indirect costs can include changes in socioeconomic and geographic inequalities depending on SFM distribution, unemployment resulting from prolonged illness, and changes in the demand for medicines resulting from the lack of confidence in the health-care system.

### Quality assessment.

An adapted version of the Consolidated Health Economic Evaluation Reporting Standards (CHEERS) checklist was used to assess the quality of reporting in the included studies.^24^ The CHEERS checklist contains 24 items with recommendations for the minimum amount of information that should be available in economic evaluations. The checklist is widely used in health economics, and is the most appropriate critical appraisal framework for the studies included in this review after screening and reference searching. Each study was assessed against the CHEERS criteria and a mark was given for each criterion not met. The 24 criteria were all taken into account to the best of our knowledge, unless they were not applicable to the study (Supplemental Table 5). This was the case, for example, of one included study in which the authors did not develop a mathematical model, so the criteria relative to the assumptions do not apply.^25^ Studies were grouped into four categories based on CHEERS checklist scores, similar to the categories used by two studies in 2011 and 2015.^26^^,^^27^ The categories were modified for use in this review; studies could be either excellent (0 point), good (1–6 points), average (7–12 points), or poor (11 points or more).†

## RESULTS

We retrieved a total of 1,078 records, which were stored in Endnote and uploaded to Covidence for screening. Once in Covidence, 157 duplicates were removed, leaving a total of 921 records. Using our eligibility criteria, 549 records were excluded and 372 were deemed appropriate for full-text screening. None of the titles and abstracts screened on the websites of Open Grey and the WHO Essential Medicines and Health Product Information Portal were deemed eligible for screening in Covidence.^17^

**Figure 1.**
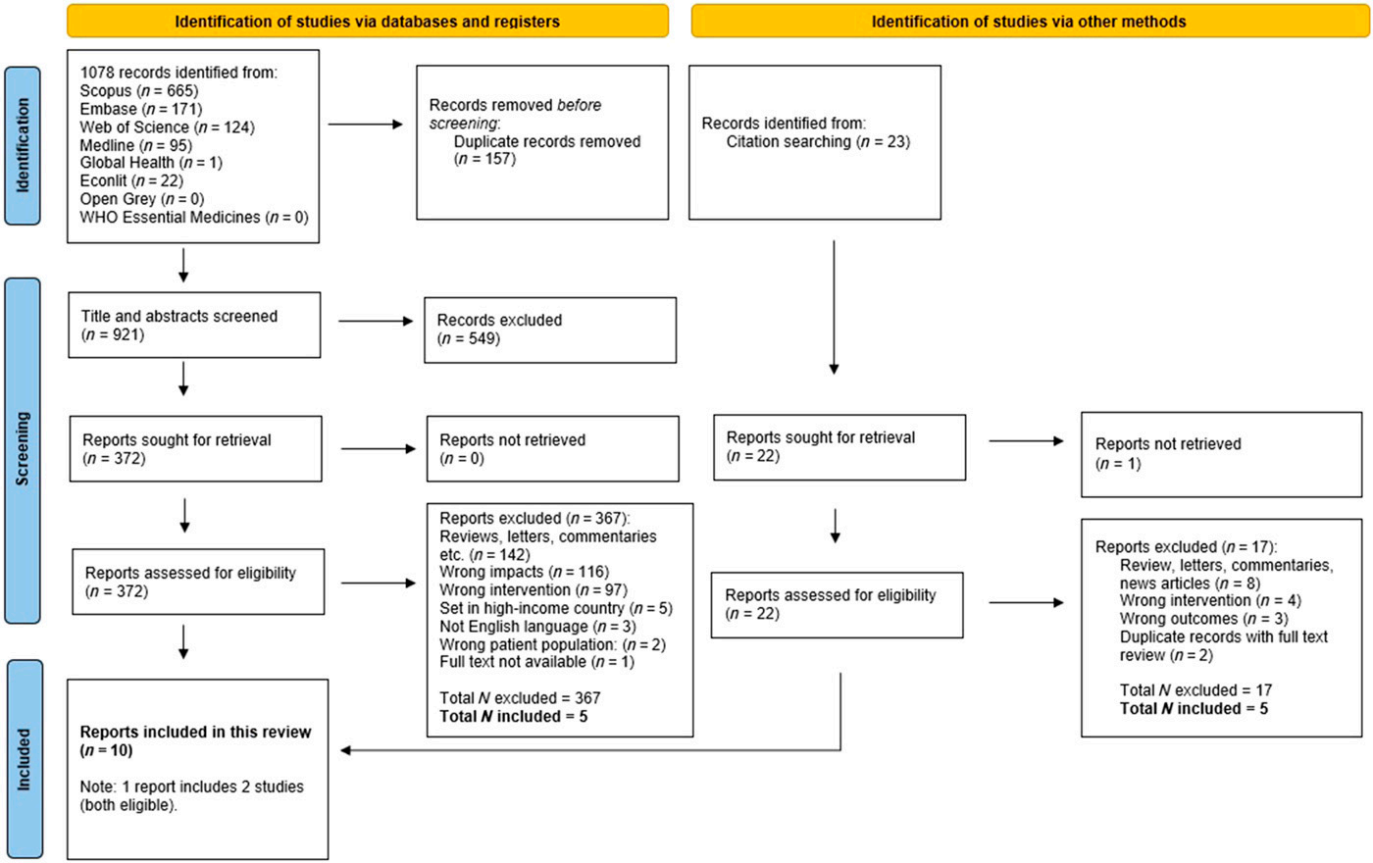
Preferred reporting items for systematic reviews and meta-analysis flowchart illustrating search and screening processes. *Source:* Page MJ et al., 2021. The PRISMA 2020 statement: an updated guideline for reporting systematic reviews. *BMJ 372:* n71. For more information, visit http://www.prisma-statement.org/.

After conducting a full-text screening, five studies were deemed eligible. In addition, we searched manually the reference lists of these five studies and conducted systematic reviews for additional studies. From the manual search, we found 23 potential matches and retrieved another five eligible studies, excluding two duplicates already identified in the database search. A total of 10 records matching our inclusion/exclusion criteria were included in this systematic review.^20^^,^^25^^,^^28^^–^^35^ One record was a report that includes two studies, both eligible, making a total of 11 studies selected.^20^^–^^22^ Figure 1 shows the details of the identification of studies through databases and citation searching.

Regarding the reports assessed for eligibility, ∼39% (142 of 367) were excluded because they were not original research (e.g., reviews, letters, commentaries).^7^^,^^8^^,^^12^^–^^14^ An additional 213 articles were excluded because they focused on outcomes out of the scope of our study, such as one qualitative study we reviewed.^36^ A further 213 articles were excluded because they focused on outcomes out of the scope of this study, such as one qualitative study reviewed.^36^ Literature focusing on medical adverse events fell into the exclusion criteria.^37^ Three studies were excluded for not being available in English, one for studying an animal population,^38^ and five for being set in a high-income country.^39^^–^^43^

The 11 studies included were assessed for quality of reporting using the CHEERS checklist.^23^ Quality assessment findings are presented in Supplemental Figure 1. Data extracted from the studies included are summarized in Tables 1 and 2. All studies included in our review had geographic settings in the region of SSA.

**Table 1:** Summary of studies that estimate impacts of substandard and falsified medicines

Author (year of publication)	Geographic setting	Period	Drug class	Methods	Impact indicators		CHEERS score, pt	Findings
					Health	Economics		
Ozawa et al. (2019)^34^	Democratic of Congo (Kinshasa and Katanga regions)	One-year time horizon using various data sources, including the Malaria Atlas Project 2018^44^	Antimalarials	Agent-based model	No. of malaria cases	Medication costs	3	Total economic cost of USD 60 million in Kinshasa and USD 301 million in Katanga. Introduction of better quality antimalarials would reduce hospitalizations by 35,800 in Kinshasa and 128,000 in Katanga. Artemisinin resistance could lead to 1,180 more deaths in Kinshasa and 5,100 additional deaths in Katanga
					No. of malaria deaths	Hospital costs		
					No. of malaria hospitalizations	Transportation costs		
					No. of days of malaria illness	Diagnostic costs		
					No. of malaria person-weeks	Productivity losses		
Ozawa et al. (2019)^34^	Uganda	One-year time horizon using various data sources, including the Uganda Malaria Indicator Survey 2014 to 2015^45^	Antimalarials	Agent-based model (SAFARI)	No. of malaria cases	Total economic impact	1	SF antimalarials were found to be responsible for an additional 14,000 (8%) hospitalizations and 71,000 (7%) years of life lost in Ugandan children younger than 5 years. Annually, SF antimalarials had an economic impact of USD 31 million. There were estimated productivity losses of USD 25.9 million, including caregiver productivity loss, disability, and early death
					No. of hospitalized malaria cases	Direct costs, including transportation, testing, drugs, consultation, and hospitalization		
					No. of malaria deaths	Facility costs		
					Disability-adjusted life years	Out-of-pocket costs		
					Years of life lost	Productivity losses		
Beargie et al. (2019)^28^	Nigeria (countrywide, northern and southern regions)	One-year time horizon using various data sources identified from the 2017 Lature review,^28^ including the 2015 Nigeria Malaria Survey Indicator^45^	Antimalarials	Agent-based model (SAFARI)	No. of uncomplicated malaria cases	Direct costs, including medications, transportation, and hospitalization	4	Estimate that SF antimalarials are responsible for 12,300 deaths in children younger than 5 years and costs of USD 892 million annually in Nigeria. SF antimalarials are a greater burden in the northern regions of Nigeria than the southern regions
					No. of hospitalized malaria cases	Facility costs		
					Average no. of malaria cases with neurological sequelae	Out-of-pocket costs		
					Average no. of malaria deaths	Productivity losses		
Evans et al. (2019)^29^	Uganda	One-year time horizon using various data sources, including the Uganda Malaria Indicator Survey 2014 to 2015^45^	Antimalarials	Agent-based model (SAFARI)	No. of malaria cases	Direct costs, including medications, testing, consultation, transportation, and hospitalization	4	The poorest wealth quintile has 12.7 times the number of deaths resulting from SF antimalarials than the wealthiest quintile. A total of 97.9% of deaths resulting from SF antimalarials are patients from rural populations, USD 26.1 million (7.8%) of the estimated total economic burden of SF antimalarials is paid by patients, with productivity losses of USD 23.8 million resulting from early death
					No. of malaria hospitalizations			
					No. of malaria deaths	Indirect costs, including lost wages, opportunity cost of time, productivity losses resulting from neurological sequelae, and productivity losses resulting from premature death		
					Disability-adjusted life years			
Jackson et al. (2020)^30^	Zambia	One-year time horizon using various data sources, including the Malaria Indicator Survey 2018^45^	Antimalarials	Agent-based model (SAFARI)	Average no. of malaria cases	Facility costs	1	At a prevalence of 10.3% SFMs, estimated 2,610 deaths in children younger than 5 years, costing USD 141 million. Predicted that eliminating SF antimalarials would decrease deaths in children younger than 5 years by 8.1% and save USD 8.5 million annually. Artemisinin resistance as a result of SFMs use could increase deaths by 6.3% and cost USD 9.7 million annually
					Average no. of malaria deaths	Out-of-pocket costs		
					Average no. of malaria hospitalizations	Productivity losses		
					Average no. of malaria cases with neurological sequelae			
Ozawa et al. (2020)^35^	63 LMICs	N/A	Antimalarials	Linear regression	Association between Universal Health Coverage indicators and prevalence of SFMs		5	A negative correlation was found between the prevalence of SF essential medicines and indicators of essential services coverage. Reducing the reported prevalence of SFMs by 10% would save USD 598 million in Nigeria, USD 79 million in two regions in DRC, USD 14 million in Uganda, and USD 8.3 million in Zambia
	DRC, Nigeria, Uganda, and Zambia		Antimalarials	Agent-based model (SAFARI)	No. of deaths	Cost per malaria case		
					No. of hospitalizations	Cost per malaria death		
					Disability-adjusted life years	Cost per malaria hospitalizations		
						Cost per disability-adjusted life year		

CHEERS = Consolidated Health Economic Evaluation Reporting Standards; DRC = Democratic Republic of the Congo; LMICs = low- and middle-income countries; N/A = not applicable; SAFARI = Substandard and Falsified Antimalarials Research Impact; SF = substandard and falsified; USD = U.S. dollars.

**Table 2:** Summary of studies that use methods other than the Substandard and Falsified Antimalarial Research Impact Model

Author (year of publication)	Geographic setting	Period	Drug class	Methods	Impact indicators		CHEERS score, pt	Findings
					Health	Economic		
Renschler et al. (2015)^31^	SSA	2013	Antimalarials	Latin hypercube sampling	No. of malaria deaths	–	4	Across 39 SSA countries, it was estimated that there were 122,350 deaths in children younger than 5 years as a result of SF antimalarials. A total of 41.96% of the median were deaths of Nigerian children
Brock et al. (2017)^32^	Kenya	2006	Antimalarials	Deterministic compartmental model	Total no. of malaria cases	–	1	Malaria cases increased by 776.9% when SF full-dose SP was used for treatment. Half-dose SP increased cases by 558.6%, whereas no treatment increased cases by 773.6%. The proportion of malaria-resistant *Plasmodium falciparum* was 18.4% when full-dose SF was used for treatment. A half dose increased this proportion by 13.%, whereas no treatment decreased the proportion by 5.8%
					Total proportion of SP-resistant infections in human population			
World Health Organization (2017)^21^	Globally and WHO African Region	2010	Antibiotics	Vital registration data, multicause model, single-cause model	Global excess deaths from severe acute lower respiratory infections	–	3	At an SFM prevalence of 10%, it was predicated that there are 72,430 childhood deaths resulting from pneumonia when there is reduced antibiotic activity. If there is no antibiotic activity at all in the SFM, then childhood deaths increase to 169,271
					Excess deaths from acute lower respiratory infections in the WHO African Region			
World Health Organization (2017)^22^	SSA	Model inputs from literature review of studies published between 2001 and 2016	Antimalarials	Decision-tree model of febrile disease	No. of initial antimalarial treatment fails	Additional outpatient further care costs	4	It was estimated that 2.1% to 4.9% of total malaria deaths were the result of SF antimalarials, with an additional 31,000 to 267,000 deaths per 1 million cases, depending on the case fatality rate. As a result of further treatment seeking and care, it was predicated that there is a total annual economic impact of USD 10.4 to 44.7 million
					No. of treated malaria cases that become severe			
					No. of deaths after receiving initial antimalarial treatment	Additional inpatient care costs		
					No. of disability-adjusted life years after receiving initial antimalarial treatment			
					No. of World Malaria Report cases	Cost of World Malaria Report cases		
					Clinton Health Initiative estimate of annual fevers	Cost of Clinton Health Initiative annual fevers		
Mori et al. (2018)^25^	Tanzania	2005–2015	Antibiotics, antimalarials, antiretrovirals, and others	Cost study	–	Cost of substandard medicines	3	From 2005 to 2015, the total economic cost of SFMs and cosmetics with banned ingredients was USD 1.2 million. Substandard medicines cost USD 13.7 million, falsified medicines cost USD 100,000, and cosmetics with banned ingredients cost USD 1.3 million. Many commonly used antibiotics, antimalarials, antiretrovirals, antipyretics, and vitamins were found to be substandard or falsified
						Cost of falsified medicines		
						Cost of cosmetics with banned ingredients		
						Other costs, including transportation, storage, court cases, and disposal.		

SF = substandard and falsified; SFM = substandard and falsified medicine; SP = sulfadoxine–pyrimethamine; SSA = sub-Saharan Africa; USD = U.S. dollars.

From the 11 included studies, nine covered the impact of having SF artemisinin-based combination therapy (ACT) medicines in the market.‡ Of these nine studies, eight estimated the health and economic impacts of SF antimalarials. Of the studies that did not focus on SF ACTs only, one estimated the annual costs of SF antibiotics, antimalarials, antiretrovirals, and other drugs such as aminophylline, paracetamol, diazepam, and prednisolone between 2005 and 2015.^25^ In addition, one report contained two studies estimating the impact of SF antimalarials on childhood malaria globally and in the WHO Africa Region.^20^ Specific medicines used to treat childhood malaria in these settings are not specified in the study. Because of the scarcity of reliable population-based data from LMICs, most of the included studies simulated a variety of scenarios over a range of SFM prevalence.^30^^–^^32^^,^^35^ For example, impacts of SFMs at alternative prevalences can be simulated with estimates of 10.3% and 19.1%.^30^ Most of the selected studies used modeling to estimate the impacts of SFMs, parametrizing each model with the best and most recent data available from the literature and open access databases (e.g., ACTwatch, Global Burden of Disease, Malaria Atlas Project).^28^^–^^35^ In the WHO report, an expert group was consulted to refine the parameters of the study.^30^ The group compared empirical estimates for the Kenyan population with their baseline model output to test whether the model predicted outputs within an acceptable range. More recent approaches used data on reported prevalence of SF essential medicines among LMICs gathered from a systematic literature review and meta-analysis,^47^ together with data on SFM prevalence and UHC indicators.^35^ Only one study in our review used empirical data, from a regulatory authority and the major importers and distributors of pharmaceuticals from 2005 to 2015, to estimate the impact of SFMs.^25^ Social impact associated with losing trust in the health system and workers, unemployment, or increased inequalities at regional, gender, and ethnicity levels resulting from SFM prevalence were not measured explicitly in any of the 11 studies included in our review.

The 11 studies used different types of methods to estimate the impact of SFMs. The methods varied from cross-sectional analysis to analytical models, such as a decision tree, logistic regression, agent-based model, and compartmental model.§ Six of the 11 studies used the SAFARI model, a dynamic agent-based model used specifically to estimate health and economic impacts of SF antimalarials.

An application of the SAFARI model was first published in 2019, focusing on Uganda and the Democratic Republic of the Congo (DRC).^33^^,^^34^ This, in itself, represented a novelty at the time, because most studies on this topic conducted analyses on the entire region of SSA, rather than at the national level. In the SAFARI model, agents seek care and treatment from a variety of health facilities as they move through different health states—healthy, infected (symptomatic or asymptomatic), or dead—over specific time intervals and time horizons. Model inputs typically rely on demographic and epidemiological data, care-seeking behavior, medication stock by facility, probability of stockouts of antimalarials, medication effectiveness, the average cost of antimalarials in facilities, nonmedication costs, and proportion of SFMs. Commonly used data sources for the SAFARI model inputs and parameters were the Malaria Atlas Project, ACTwatch, and the WHO World Malaria Report.^44^^,^^46^^,^^49^ The health and economic impact indicators estimated by the SAFARI model can be found in Table 2. The model has also been applied to measure the impact of specific interventions. As an example, SAFARI has been used to evaluate an intervention that replaced all antimalarials with good-quality ACTs in Zambia.^30^ Results showed that the economic impact of SF antimalarial medicines accounted for U.S. dollars (USD) 31 million—8% of the total economic impact of malaria among children who sought medical care. Applying the same methods to the DRC,^34^ SFMs were estimated to cost ∼35% of total annual malaria costs in Kinshasa Province (USD 20.9 million) and 43% in the Katanga region (USD 130 million). These costs were even greater when the model accounted for the possibility of patients developing resistance to the active substance by consuming SFMs.

The authors of the SAFARI model followed up with the evidence found in Uganda and the DRC and, in a study published in 2020 they investigated the relationship between UHC indicators and the prevalence of SFMs in different countries of SSA.^35^ This was a novel application of the SAFARI model, in which they analyzed different scenarios of possible policies designed to improve health-care provision in each country.^35^ Investing in quality improvement of antimalarials by 10% was estimated to result in annual savings of USD 8.3 million in Zambia, USD 14 million in Uganda, USD 79 million in two DRC regions, and USD 598 million in Nigeria (excluding the intervention costs).

The SAFARI model has also been used to show that SFMs affect rural and low-SES populations disproportionately.^29^ Researchers have found that those in the lowest SES quintile were 23 times more likely to have been hospitalized as a result of SF antimalarials than the highest SES quintile. Investigating the impact of SFMs on specific subgroups is key in identifying particularly vulnerable populations that can be targeted by specific policies.^45^ Studies using methods other than the SAFARI model are listed in Table 3.

**Table 3:** Main conclusions and recommendations

Conclusion	Recommendation
SFMs may affect rural and low-socioeconomic status populations disproportionally	Researchers and national authorities should collect data and analyze impacts on specific subgroups to be targeted by policies
More and better observational data could improve researchers’ capacity to analyze the impacts of SFM availability	Following WHO guidance, national regulatory authorities should be responsible for recording data and implementing after-market surveillance systems to create appropriate partnerships and share information at the international level, complying with all ethical considerations and protecting the privacy of all agents involved
Existing evidence on the health and economic impact of SFMs lacks coverage at the geographic level and with regard to medicine classes. We could not find any studies that measure the social impact of SFMs	Researchers should develop and disseminate methods with clear guidelines for measuring the health, social, and economic impacts of SFMs to be used as a benchmark for similar studies in different contexts and for looking at different substances
Some existing models are complex, data demanding, and arguably difficult to be implemented at the global level by public service institutions	Researchers should provide tool kits and more methodological guidance on designing and conducting substandard and falsified impact studies, and develop user-friendly tools (i.e., low cost and easy to replicate) to motivate the implementation of monitoring activities, without overloading frontline services

SFM = substandard and falsified medicine.

The first study included in our review aimed to estimate any impacts of SFMs in LMICs.^31^ The authors used an uncertainty model with Latin hypercube sampling (LHS) to estimate the number of deaths in children younger than 5 years in SSA resulting from the consumption of SF antimalarials. Latin hypercube sampling uses “sampling without replacement”—a method used to simulate a sample that reflects the distribution of the population.^50^ After 10,000 calculations performed using LHS, the study estimated that, in 2013, there were more than 120,000 deaths in children younger than 5 years resulting from SF antimalarials across 39 countries in SSA, with Nigeria accounting for 60.6% of the deaths.^50^ Based on 2010 WHO estimates of under-five malaria deaths, this number represents about 22.33% of total deaths.^31^

In terms of economic costs, we found estimates of annual expenses associated with the presence of SFMs in the market.^34^ Data were sourced originated from the Tanzanian regulatory authorities, importers, and distributors of pharmaceuticals, with information covering the period of 2005 to 2015.^32^ Using a simplified “ingredient approach,” the authors calculated that, during the study period, substandard medicine costs to the national government were ∼USD 13.65 million and falsified medicine costs were USD 149,369.^32^ In this study, the prevalence of substandard medicines had a considerably greater economic impact than falsified medicines in Tanzania.^32^ The data were extracted from confiscation reports held by the regulatory authority, with information on SFMs collected from 2005 to 2015. The ingredient approach consists of identifying, quantifying, and validating individual items. Cost estimates were then computed by multiplying the tallied quantities with unit prices for each item using the median buyer prices from the International Drug Price Indicator Guide or the Tanzanian Medical Stores Price Catalogue of 2015 to 2016,^32^ depending on the data availability.^32^ The retrieved data also allowed the identification of SFM manufacturers in the national market, which can be a helpful tool in other geographic settings as well.

Decision tree models, frequently used in cost-effectiveness studies, have also been applied to estimate the impact of SFM prevalence in the market. As part of a WHO 2017 report^22^ on this subject, one study used a cost-effectiveness approach to investigate the impacts of SF antimalarials on childhood malaria in SSA.^22^^,^^51^ According to the estimates, the use of antimalarials with an active pharmaceutical ingredient of less than 85% caused ∼529 deaths per 1 million malaria cases. In the same report, there is another relevant contribution to this subject that studies the impact of SF antibiotics on under-five mortality resulting from childhood pneumonia.^21^ The method adopted was a multinomial logistic framework using vital registration and data-based multi- and single-cause models in India and China.^52^ The authors analyzed different scenarios to estimate the change in the number of deaths associated with a change in the prevalence of SFMs.^52^ For example, simulating a scenario of 1% prevalence of SF antimicrobials, the authors estimated that the pneumonia case fatality rate would double, and the number of deaths in children younger than 5 years would be approximately 8,688; a 10% prevalence would result in an estimated increase of 72,430 in under-five mortality in that scenario.^21^

One study from 2017 used a deterministic compartment model that involves a human–mosquito system to quantify the impact of sulfadoxine–pyrimethamine (SP) quality on the transmission of SP-sensitive and SP-resistant *Plasmodium falciparum* between humans and the female *Anopheles* mosquito in Kenya.^32^ Compartment models divide the population into susceptible, infected, and recovered from the disease, and then estimate how people fluctuate over time among the defined compartments.^32^^,^^51^ In this study, humans can be either susceptible or exposed to SP-sensitive and/or SP-resistant *P. falciparum*, whereas the female *Anopheles* mosquito can be either susceptible, exposed, or recovered from *P. falciparum*.^32^ These compartments are defined by time‐varying functions with no random fluctuations, determined by the parameters and initial conditions. Sulfadoxine–pyrimethamine is no longer recommended for the treatment of malaria because of widespread SP resistance; however, it is still prescribed commonly in Kenya.^32^^,^^49^ The authors showed that the use of SF and subtherapeutic doses of this medicine increased the numbers of human malaria cases and SP-resistant infections by 776.9%.^32^

The studies examined in our review show that methods used to measure the impact of SFMs have evolved relatively quickly during the past decade, going from a simple uncertainty model using LHS in 2015 to the SAFARI agent-based model in 2019. This progress results in an increase in model complexity that brings researchers closer to simulating the reality of how medicine quality affects patient outcomes and disease progression. The earliest studies use methods that were more static, with more demanding assumptions. The LHS used with a simple uncertainty model produced general estimates of the number of deaths resulting from SFM prevalence, but it did not allow for simulating how SFM prevalence can affect patient behavior and disease progression, nor did it allow for individual specific variations.^29^ The same is true for the costing study.^25^ This is a simple method of identifying and summing up costs, which is only suited to measure tangible costs, and it does not include any simulation. To overcome the restrictions of these simpler methods, produce projections over time, and test potential interventions, other studies included in this review used analytical models to examine the impacts of SFM prevalence. The decision tree model, for example, is used to simulate decision processes when an event or choice results in a set of potential outcomes with different probabilities of happening.^24^ Another study used a multinomial logistic regression to predict the impact of one or more independent variables (including SFM prevalence) on a specific outcome variable (child mortality) with more than two categories, but results can be more difficult to interpret than those of the decision-tree model.^21^ Although the latter two methods can measure, to some extent, the different ways that SFMs can affect disease progression and survival, as the length of the study and the number of events or decisions increase, these can quickly become computationally overwhelming and difficult to follow.^53^ In turn, compartment and agent-based models can deal with most of these computational limitations. The compartmental model study included our review quantifies the transmission dynamics between mosquitoes and humans, and measures how medicine quality affects those dynamics and malaria transmission.^32^ Agents in these models are grouped and treated as interchangeable, so they cannot be used to study individual behavior.^54^ In turn, agent-based models can grasp complex decision processes and, by allowing one to define agents and states, can produce a representation that is closer to reality. The SAFARI model in particular was developed and adapted specifically to measure how SFMs can affect populations at different levels.^33^^–^^35^ The SAFARI model accounts for patient-specific as well as national and regional variation, and allows researchers to specify costs and financing sources.^33^^–^^35^ Evidence has shown that compartmental and agent-based models can both be used to model transmission routes and can even produce similar results, but the latter allows for studying individual, specific variation through time, which gives further information.^55^ One potential and relevant drawback of agent-based models such as SAFARI is the requirement of multiple types of high-quality data, as well as software, mathematical modeling expertise, and time. These factors can represent disincentives for national authorities to read, trust, and replicate the analysis in other contexts or to promote the study of the impacts of SFM prevalence in general.^56^

## DISCUSSION

This is the first systematic review to identify the methods used to estimate impacts of SFMs and outline the geographies and drug classes for which this has been done. This review acts as a resource for those who aim to work on SFM prevalence impacts by making an updated, comprehensive collection and analysis of the most relevant contributions to this subject and thus outline the current gaps in the literature. The first objective of our systematic review was to identify the methods used to estimate the health, economic, and social impact of SFMs in LMICs. More than half the included studies (*n* = 6) used the SAFARI model for estimating SFM impacts.^28^^,^^29^ Other methods included a simple uncertainty model using LHS, a costing study, a decision tree model, and a deterministic compartmental model.^50^^–^^54^ Estimates indicate that SF antimalarial medicines can account for 10% to ∼40% of total annual malaria costs. Results also showed that SFMs affect rural and low-SES populations disproportionately, and thus it is key to analyze the impacts on specific subgroups that should be targeted by policies.^25^^,^^28^^–^^35^

Our second objective was to identify the current gaps in the literature in terms of geographic setting, medicine classes, and impact. Namely, all included studies determined the health and economic impacts of SFMs only in the region of SSA, leaving all LMICs in other regions out of this review, as well as several classes of medicines. Nine of the 11 studies estimated the impacts of SF antimalarials only, one report included SF antibiotics,^20^ and another estimated the impact of a range of SFMs, including antibiotics, antimalarials, and antivirals.^32^ An important gap identified is that we could not find any study that measured the potential social impact of SF prevalence, and there is a lack of data regarding health outcomes (after treatment with medication) as well as costs associated with post-treatment health care.

Existing literature has shown that LMICs bear most the burden of SFMs, likely a result of poor surveillance mechanisms, governance, regulations, and management of pharmaceutical supply chains.^36^ Corruption, weak governmental policies, and limited technical capacity can also be responsible for enabling the distribution of SFMs in LMICs and decrease the likelihood of detecting SFMs.^36^^,^^57^ Governments in many LMICs are striving to achieve UHC, which may result in political pressure to decrease drug prices so they are accessible to patients from all levels of economic status. This may jeopardize medicine quality.^36^^,^^57^^,^^58^ High-income countries are also affected by the distribution of SF drugs. Between 2005 and 2010, there was a reported 92% increase in falsified drug sales in the United States amounting to a total of USD 75 billion.^58^ Although the previously mentioned risk factors are common to several countries around the world, our review shows that most of the existing studies estimating the impact of SFMs were developed in the region of SSA only. To understand the real magnitude of the prevalence of SFMs, future studies on this subject must expand geographic coverage; otherwise, the evidence will always be limited by the specificities of a single region.

Studies of SFMs have not only been focusing on the region, but also on the class of medicine, which has been mostly antimalarials and antibiotics. Conditions treated commonly with antibiotics are a great burden on LMICs, and there is evidence of antibiotics and antimalarials being targets of SFM production.^6^^,^^47^^,^^59^ As for antimalarials, after 2001, when the WHO declared ACTs as the first-line medicine for malaria treatment,^46^ reports of various surveys in Southeast Asia showed that up to 50% of the artesunate monotherapy sold was fake, and studies predicted this situation could get worse.^60^^,^^61^ This, together with the presence of ineffectual drug regulation and inadequate technical capacity in many LMICs with endemic malaria, create a big challenge for the development of effective policy action on SFMs.^62^ In 2018, Ozawa et al.^6^ found that 19.1% of antimalarials and 12.4% of antibiotics were substandard or falsified. Although these can be potential reasons for SF antimalarials and antibiotics being the focus of most SFM studies, WHO Global Health estimates show that it is not always well justified.^59^ In 2019, although most of the top 10 diseases to cause death in low-income countries were indeed communicable diseases, noncommunicable diseases such as ischemic heart disease and stroke ranked third and fourth, respectively.^59^ In LMICs, ischemic heart disease and stroke are the number-one and -two causes of death.^63^ Malaria was the sixth common cause of death, claiming 190,000 lives in 2019, whereas 416,363 people died of lower respiratory infections and 262,905 resulting from diarrheal disease.^63^ These data suggest there is a need for more research on medicines used to treat noncommunicable diseases such as diabetes or hypertension, diseases with an increasing burden over time, and essential medicines, including cough syrups, antiepileptics, or anesthetics, for which there are historical records reporting cases of falsification or other types of corruption, with dramatic consequences for their users, often children.^59^^–^^65^

Access to effective and safe, essential health care for all is a public health priority, and now even more topical because of the ongoing COVID-19 pandemic. In March 2020, U.S. Customs and Border Protection seized fake COVID tests and the World Customs Organization reported an increase in falsified hand sanitizers and respirator masks.^66^^,^^67^ Falsified chloroquine, reported by some in the media to be a remedy for COVID-19, was discovered to be in circulation in West Africa.^68^ Substandard and falsified medication can lead to an increase in the number of adverse events, and patients may become even more reluctant to seek health-care treatments, further amplifying the impacts of SFMs, particularly severely on advanced diseases.^14^^,^^69^ Patients may also become disillusioned by generic brands and may purchase expensive brand-name medicines to avoid SFMs.^14^^,^^69^ This is a pertinent issue particularly for many LMICs, where out-of-pocket costs for medical treatment impoverish ∼90 million people annually.^2^^,^^5^ On the occasions that governments provide free or subsidized medicines to patients, public authorities are affected directly by the presence of SFMs in the market.^7^^,^^14^^,^^64^^–^^67^ The methods identified in our review show progressive and heroic efforts to grasp the broadness of SFM prevalence impacts. There is a clear evolution in the analytical methods adopted over a relatively short period of time that improves the capacity to quantify impacts and study the different channels through which SFMs affect populations and governments. Although our review focused on quantitative studies, the use of qualitative methods has made extremely relevant contributions to this field—namely, related to the potential economic and political factors that enable the presence of SFMs in the market.^36^ This suggests that a more interdisciplinary approach using mixed qualitative and quantitative methods can help our understanding of the effects of SFMs on all involved parties, from patients to health-care professionals, manufacturers, and government institutions.^36^^,^^57^

The findings of our systematic review generally match the results from one earlier review on the methods to measure impacts of SFMs.^70^ That study, by Ozawa et al.^70^ from mid 2022, identified 9 of the 11 studies in our review. The authors highlight the roles of simulation models in providing estimates of the impact of SFMs, and the lack of data on the costs and effectiveness of interventions to improve medicine quality provision and monitoring.^70^ Our study goes beyond what has already been published by including a review on the measurement of social impact. In fact, one of our main conclusions is that we could not find any studies that cover these impacts, despite their relevance to the design of health-care policies, which is a valuable and new contribution to the literature. In addition, our review further supports and complements the earlier findings by providing a systematic analysis of the existing literature, using quality checklists, and going into more detail on the current evidence and existing methods. This more detailed approach also allowed us to explore further the gaps found in terms of primary data collection and availability, and the need to expand studies on the impact of SFMs to other regions and more medicine classes.

Moving forward, this review endorses WHO recommendations to improve the estimation of public health and socioeconomic impacts of SFMs that there is a need to adopt more consistent standards for data collection, analysis, and reporting of SFM impacts.^20^ Consistency in the definitions of SFMs used on guidance and testing of suspected SF medical products can allow for more replicability and comparison across studies, which would improve our understanding of the nature and magnitude of the issues associated with the prevalence of SFMs. This would naturally contribute to close the main literature gaps found in our systematic review. In addition, there is a need for more primary data collection and availability (e.g., patient characteristics before and after treatment, laboratory results) that would allow for more robust estimates of the prevalence of disease, behaviors, or SFMs. According to the WHO, this could be achieved by having national regulatory authorities responsible for recording data and implementing after-market surveillance systems to create appropriate partnerships to share information at the international level.^20^ We also consider that the existence of user-friendly tools to measure SFM impacts (i.e., low cost and easy to replicate) can be important to help and incentivize governments to monitor how SFMs are affecting their populations with methods they can trust, without overloading their capacity.^55^

Prior to 2006, it was widely accepted that, globally, 10% of drugs were counterfeit.^8^^,^^9^ In that same year, the International Medical Products Anti-Counterfeiting Taskforce published a document updating the estimates and explaining that using a blanket figure of 10% worldwide was reductive.^71^ More than a decade has passed and the WHO’s estimation on the prevalence of SFMs in LMICs is at 10.5%. To our knowledge, all currently available SFM prevalence estimates were not derived from laboratory trials (i.e., from medicines collected in the field and subject to laboratory testing), but from applying several strong assumptions to aggregated data, which causes a large uncertainty about the real prevalence and impacts of SFMs.^72^^,^^73^

Our systematic review has several limitations. Because all studies included in this review were developed in SSA, our findings may not be generalizable to other settings. Considering that the evidence is concentrated in six countries in SSA (Uganda, Nigeria, Zambia, DRC, Kenya, and Tanzania), the findings may not be applicable to other LMICs or even to other African countries, because of the socioeconomic differences among countries and quality of data sources.^62^ Our search was also limited by the fact that, to our knowledge, the existing evidence on the impacts of SF medical products does not include vaccines or diagnostic kits.^20^ Because there are limited data on the prevalence of SF antimalarials and great variation in estimated prevalence, the findings included in these studies may be underestimations. For example, one study used a prevalence of 19.1% of SF antimalarials in the DRC, while other studies have reported the prevalence of SF antimalarials to be as high as 62% in Kinshasa.^33^^,^^64^ In addition, the studies included in our review did not estimate the costs of implementing interventions to reduce the prevalence of SFMs. The studies found estimate costs of SFMs and changes attributed to the interventions, but do not count for how much the interventions themselves cost, which is extremely relevant for policy implementation purposes.

In conclusion, the development of a standard method for measuring the health, social, and economic impacts of SFMs can improve the existing coverage of geographic regions, SF drug classes, and population characteristics. Moreover, even though mathematical models have allowed us to grasp some of the magnitude of this problem, better observational data could improve researchers’ capacities to analyze the effects of SFMs. Having better primary data to feed the analytical models would allow for more in-depth and robust evidence on this subject. At the same time, acknowledging the importance of complying with all ethical considerations when developing observational studies related to health care and improving SFM monitoring systems (including alerts, sampling, collection, testing, and identifying health impacts) would bring a valuable contribution to the existing literature.^72^^,^^73^ Table 3 provides a summary of the main conclusions and respective recommendations that resulted from our study. We discovered that mathematical models such as SAFARI are currently the most used approach to estimate the effects of SFMs in LMICs. We found several important gaps in the literature, such as the lack of studies on the impacts of SFMs in LMICs outside the region of SSA, the lack of coverage of different medicine classes (other than antibiotics and antimalarials), and the lack of evidence on the potential social impact of SFM prevalence. More methodological guidance on designing and conducting SF impact studies is needed for a more nuanced understanding of this complex topic.

## Supplemental Material


Supplemental materials

## Footnotes

† The scale used by two reference papers was reversed for the purpose of our review because some of the CHEERS criteria was not applicable to some studies.^18^^,^^19^ Allocating points according to criteria missed allowed a fair comparison among studies.

‡ Artemisinin-based combination therapies are the first-line therapy in almost all countries where malaria is endemic because of its high efficacy and tolerability, and ability of artemisinin-based combination therapies to reduce ongoing transmission of the parasite.^46^

§ According to the literature, agent-based models “allow one to simulate the individual actions of diverse agents, and to measure the resulting system behavior and impacts over time.”^48^
